# Computational Analysis of Nonuniform Expansion in Polyurethane Foams

**DOI:** 10.3390/polym11010100

**Published:** 2019-01-09

**Authors:** D. Niedziela, I. E. Ireka, K. Steiner

**Affiliations:** Department Flow and Material Simulation, Fraunhofer Institute for Industrial Mathematics, Fraunhofer-Platz 1, D-67663 Kaiserslautern, Germany; iirex4@gmail.com (I.E.I.); konrad.steiner@itwm.fraunhofer.de (K.S.)

**Keywords:** polyurethane foams, nonuniform expansion, foam fraction distribution, reaction injection molding, chemorheology, finite-volume method

## Abstract

This paper computationally investigates heterogeneity in the distribution of foam fraction in chemically expanding blown polyurethane foam. The experimentally observed disparity in the volumes of expanded foam when an equal mass of the foaming mixture was injected into tubes of different dimensions motivated this study. To understand this phenomenon, attributed to local variations in the thermal and rheological properties of the expanding system, we explore available data from free-rise foam-expansion experiments in different geometries. Inspired by the mathematical framework for the microstructure modelling of bubble growth in viscous liquids, we study the reacting mixture as a continuum and formulate appropriate mathematical models that account for spatial inhomogeneity in the foam-expansion process. The nonlinear coupled system of partial differential equations governing flow was numerically solved using finite-volume techniques, and the associated results are presented and discussed with graphical illustrations. The proximity of the foaming-mixture core to the external environment and the thickness of a thermal-diffusion layer formed near the bounding geometry was seen to influence the distribution of the foam fraction. Our simulations showed an average spatial variation of about 1.1% in the distribution of solid foam fraction from the walls to the core, as verified with data from μCT scan analysis of the expanded foam. This also reflects the distribution of void fraction in the foam matrix. The models were validated with experimental data, and our results favourably compared with the experiment observations.

## 1. Introduction

The commercial relevance of flexible or rigid polyurethane (PU) foams has stimulated extensive research interest in their production and product-optimization processes. In their production phase, PU foams exhibit complex behavior initiated by premixing relevant isocyanate and polyol groups in the presence of suitable catalyst and blowing agents. Depending on the type of blowing agent, among many factors affecting the foaming process [1], the resulting PU foam matrix is classified as rigid or flexible foam. However, certain factors, such as mixture rheology, amount of nucleated bubbles in the mixture, rate of depletion of the created/injected gas, or other external control mechanisms, affect their expansion process.

In flexible foams, the reaction between isocyanate and water in a chemically blown system results in the formation of amine and CO${}_{2}$ gas. This CO${}_{2}$ gas diffuses into nucleated bubbles in the mixture due to a pressure difference [2], leading to a continuous increase in mixture volume until the reacting water is totally converted. More so, the combined effect of the evolution of mixture viscosity via chain-linking/polymerization, urea formation, as well as bubble rupture also limit the expansion process. This process results in a foam matrix with open cells [3]. On the contrary, physical blowing agents, such as trichlorofluoromethane, cyclopentane, or liquid CO${}_{2}$, do not react in the mixture [4]. However, due to their relatively low boiling point, the associated hydrocarbon vaporizes (at its boiling temperature) as a result of the exothermic nature of the foaming system [5]. The vaporized gas diffuses into the nucleated bubbles in the mixture, leading to the expansion of the reacting mixture and the formation of a foam matrix with closed cells and rigid morphology

The PU foam expansion process may be homogeneous or heterogeneous depending on whether the system is controlled or not. This, in turn, influences pore structure and pore-size distribution [6], as well as the thermophysical and physicomechanical properties of the final foam matrix [7,8,9,10]. However, some applications of PU foams require homogeneously distributed bubbles in the final product. Situations arise, for example, in a geotechnical engineering process, where the expanding system cannot be controlled, thereby resulting in a heterogeneous expansion of the foaming mixture [11]. Such inhomogeneity in bubble-size distribution, attributed to the spatial variation of flow properties, results in a subdivided domain consisting of zones where the bubbles grow freely, and zones where their growth is restricted [7,12].

Several successful attempts to predict bubble growth and their size distribution in various liquids from a microscopic (cell model) view have been reported in the literature [7,8,9,13,14,15,16,17,18]. For instance, the theoretical modelling and analysis of the evolution of a vapor bubble expanding in a shell of power-law (non-Newtonian) fluid by Street et al. [13] illustrated the influence of the shear thinning, melt viscosity, and molecular diffusivity of the blowing agent on the initial growth of the bubble. In addition, the mass and momentum transport process of the fluid was also shown to significantly affect the initial growth rate of the nucleated bubble [13]. In a related study [7], the effect of blowing-agent concentration and gelling (cream) time on the expanding bubble in a PU foam mixture, as well as bubble-size distribution, was presented. To understand the influence of fluid viscoelasticity on expanding bubbles, Feng and Bertelo [8] carried out an extensive study on bubble expansion in a viscoelastic (Oldroyd-B) fluid. Adopting the cell model, they simulated and discussed the effect of gas depletion and the proximity of neighboring bubbles in the physically blown polymer, and predicted the evolution and size distribution of the bubbles in reasonable agreement with available experimental data.

In their work, Amon and Denson [14] presented a mathematical framework for a system of expanding bubbles, with each bubble enclosed in a shell of liquid containing supersaturated gas. Relevant features that allow for the possible extension of the proposed model to the macroscopic investigation of bubble growth in liquids were analyzed. Based on the cell model [13,14] and neglecting surface tension effects, Bruchon and Coupez [15] numerically tracked the evolution of the radius of a single bubble in a Newtonian fluid and further carried out 2D and 3D simulations of a finite number of bubbles expanding in a pseudoplastic fluid. Although the cell model provides relevant qualitative information on bubble dynamics in liquids [14], it reduces the foam-structure formation to the resolution of tracking the evolution of the radius of a limited number of bubbles within the liquid [15]. This becomes more complicated and (numerically) expensive when an increasingly large number of bubbles [15,16], as in the case of PU foam formation, expands heterogeneously under nonisothermal conditions.

Motivated by the theoretical framework in References [13,14,15] and the challenge in Reference [15], we sought to understand and predict the nonuniform expansion observed in a thermally uncontrolled PU foam-formation process. We propose a macroscale (continuum) model that accounts for local variations in the foam-expansion process. We adopted the modelling approach of Reference [19], which summarizes the specie consumption with the Kamal law [20] for the degree of cure, with an adequately modified expansion source term and accounting for local contributions of the mixture temperature and viscosity to the expanding system. Although this approach does not quantify the bubbles in the foam, it gives a qualitative description of the distribution of void fractions in the domain. Of particular importance in this study was to investigate and understand the observed volume variation of the expanded foam when an equal mass of the reacting foam was injected into different cylinders in free-rise experiments. In this regard, three foam-expansion experiments in different geometries were studied and simulated. With graphical illustrations, we present our results and validate them against available experimental data. Furthermore, we carried-out μCT scan analysis of the expanded foam matrix and compared our observations with results from our simulation. Our results show qualitative agreement with observations from the experiments.

The rest of this paper is structured as follows. In Section 2, we briefly describe the experimental setup and present a mathematical framework for the nonuniform expansion source term adopted in our simulations. The results and corresponding discussions are presented in Section 3, and we conclude the study in Section 4.

## 2. Experiment and Mathematical Framework

**Figure 1:**
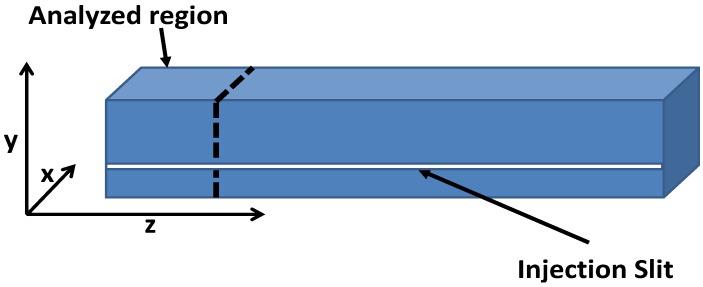
Schematic description of the geometry of the closed rectangular mold in the reaction injection molding (RIM) experiments. The marked region corresponds to the part of the expanded foam analyzed with μCT scans, (see Section 3.2).

Following the experiment and discussions in Reference [19] on the free-rise PU foam-expansion process, equal masses (77 and 37 g) of a reacting mixture containing isocyanate and precursors for rigid foam with some water are injected into different cylindrical tubes in a series of experiments at room temperature. Each pair had a diameter of 56 and 112 mm, and a height of 812 and 203 mm, respectively. In related reaction injection molding (RIM) experiments, the same mixture was injected into a rectangular mold of 500 × 50 × 40 mm (Figure 1) under a fixed wall temperature of 55 ${}^{\circ }$C. The expanding foam in each tube was monitored, and their heights and volumes were recorded in time with the aim of understanding the effect of the mold or geometry conditions on the expanding PU-foam system, particularly since the free-rise experiments in the cylinders resulted in a foam matrix of different volumes. This suggested a possible influence of the geometry conditions on the foam-expansion process.

Assuming that the foaming mixture is a quasihomogeneous continuum with a constant rate law for the degree of cure, the experiments presented above were modelled mathematically, subject to appropriate boundary conditions. The equations governing the transport of mass, energy, and degree of polymerization follow directly from Reference [19] (see Appendix A). However, the source term in the equation governing the conservation of mass is adequately restructured to account for possible nonuniformity in the expanding system.

The mathematical formulations of Street et al. [13], and Bruchon and Coupez [15] from the conservation of mass and momentum transport describe the dynamics of a bubble of radius *R* expanding in a pool of viscous liquid under quasistatic motion. Adopting the continuity of stresses at the surface of the bubble and neglecting the surface-tension effect, the evolution of the bubble radius was summarized by:

(1) $$\frac{\dot{R}}{R}=\frac{P_{b}-P_{l}}{4\mu_{l}^{eff}}.$$

where $P_{b}$ and $P_{l}$ are the pressure in the bubble and liquid, respectively. $\mu_{l}^{eff}$ is the effective viscosity of the surrounding liquid, and $\dot{R}$ is the rate of change of the radius of the bubble as it expands in time. Equation (1) implies that, for a given bubble, expanding in a liquid with viscosity $\mu_{l}^{eff}$ under isothermal condition

(2) $$P_{b}-P_{l}\propto \mu_{l}^{eff}\frac{\dot{R}}{R}.$$

The equation governing the conservation of mass of the expanding PU foam relates flow velocity v to foam volume in time (V(t)) (see Reference [19]) by:

(3) $$\nabla \cdot v=\frac{1}{V(t)}\frac{dV(t)}{dt}=S_{p}.$$

$S_{p}$ here is the expansion source term. We adopted a growth model [19,21,22] for the volume of the expanding foam, so that at any time *t*, foam volume V(t) is described by

(4) $$V\left(t\right)=A\exp\left(-\bar{\pi }\left[{\left(t+t^{*}\right)}^{-\bar{\varepsilon }}\right]\right)+\gamma ,$$

where $t^{*}$ is the time when the foam starts to expand after being injected into the mold. Constants *A*, $\bar{\varepsilon },\bar{\pi }$, and γ are estimated from available volume-expansion experiment data.

In a thermally controlled (adiabatic) foam-expansion setup, the source term, denoted here as $S_{p}^{ad}$, is ideally obtained from volume V(t) of the expanding PU foam under spatially uniform temperature conditions. Consequently, this results in the homogeneous expansion of the foaming mixture at constant pressure. Hence, suppose there are *n* nucleated spherical bubbles in such an expanding system, each bubble with an *R* radius, then one can show that the contribution of $S_{p}^{ad}$ to individual bubbles relate to the radius of each bubble by

(5) $$\frac{1}{V(t)}\frac{dV(t)}{dt}\equiv {\left(\frac{\dot{R}}{R}\right)}_{add}=\beta S_{p}^{ad},$$

where β=1/3 (see Appendix B). Therefore, under adiabatic conditions, Equation (1) can be rewritten as:

(6) $$P_{b}-P_{l}=4\beta \mu_{l}^{ad}S_{p}^{ad}$$

Here, $\mu_{l}^{ad}$ is the mixture viscosity under adiabatic temperatures.

Thermal conditions in chemorheological fluids significantly affect the rate of reaction, which has direct consequence on the evolution of the chemoviscosity of the reacting fluid [19,23]. More so, in a thermally uncontrolled (nonadiabatic) foaming system, the temperature of the expanding foam spatially varies. Under this condition, temperature measurements at the core is often approximated to be adiabatic since temperatures in this region are less diffusive. However, beyond a progressive thermal diffusion layer in the region close to the boundaries, temperature conditions are nonadiabatic. Hence, expanding bubbles in such regions would lead to restricted growth due to the combined effect of temperature, reaction rate, and mixture effective viscosity ($\mu_{l}^{eff}$) [7,12].

Dividing both sides of Equation (6) by $4\mu_{l}^{eff}$ and assuming that the pressure difference between the bubble and the surrounding liquid is locally the same under adiabatic or nonadiabatic temperature conditions, we have:

(7) $$\left[\frac{P_{b}-P_{l}}{4\mu_{l}^{eff}}=\frac{\dot{R}}{R}\right]\equiv \beta S_{p}=\frac{4\beta \mu_{l}^{ad}S_{p}^{ad}}{4\mu_{l}^{eff}}.$$

Therefore, considering local thermal influence on the growth of individual bubbles reflected in the local viscosity of the expanding PU foam, we define a linearly averaged local effective viscosity $\mu_{l}^{eff}$ by

(8) $$\mu_{l}^{eff}=\left(1-\alpha \right)\mu_{l}^{ad}+\alpha \mu_{l}^{nad},\;\;\;\;\mathrm{with}\;\mu_{l}^{nad}\geq \mu_{l}^{ad}.$$

where $\mu_{l}^{nad}$ is the viscosity of the foaming mixture under a nonadiabatic condition, and thermal sensitivity parameter α is to be determined (see Section 3.3.1 for details). Hence, Equation (7) becomes:

(9) $$\beta S_{p}=\frac{4\beta \mu_{l}^{ad}S_{p}^{ad}}{4(\left(1-\alpha \right)\mu_{l}^{ad}+\alpha \mu_{l}^{nad})},$$

Rearranging Equation (9), we obtain:

(10) $$S_{p}=\frac{S_{p}^{ad}}{1+\alpha \left(\frac{\mu_{l}^{nad}}{\mu_{l}^{ad}}-1\right)}.$$

The equation governing mixture viscosity $\mu_{m}$ as in Reference [19] is given by

(11) $$\mu_{m}=\mu_{oo}\exp\left(\frac{E_{\mu }}{RT}\right)\cdot H\left(\zeta \right)\cdot F\left(\varphi_{g}\right),$$

where $\mu_{oo}$ is constant, $E_{\mu }$ is the foam activation energy, *T* is the temperature, and *R* is the rate constant. H(ζ) and $F(\varphi_{g})$ are, respectively, the contributions of the degree of cure/polymerization ζ and the absorbed gas fraction $\varphi_{g}$ to the viscosity of the mixture. We assume for simplicity that these contributions are locally uniform so that by substituting Equations (11) in Equation (10), and simplifying the resulting expression, we obtain

(12) $$S_{p}=\frac{S_{p}^{ad}}{\Gamma_{\mu }}$$

where local damping factor $\Gamma_{\mu }$ is given by

(13) $$\Gamma_{\mu }=1+\alpha \left(\exp\left(\frac{E_{\mu }}{R}\left(\frac{1}{T^{nad}}-\frac{1}{T^{ad}}\right)\right)-1\right)$$

This implies that nonlocal parameter α controls the influence of temperature, at a constant reaction rate, on the expansion of the foam. Therefore, at any thermal conditions, the right-hand side of Equation (3) is adequately replaced with Equation (12).

In the next section, we present and discuss the results obtained from both the experiments and our simulations. Further comparison against the experimental data are carried out.

## 3. Results and Discussion

We commence this section by presenting some observations from the experimental data and follow this up with the results from our simulations. Details of the numerical method adopted in resolving the system of nonlinear partial differential equations governing the expansion process are presented in Reference [19].

### 3.1. Experiment Observations: Free-Rise Foam Expansion in the Cylinders

**Figure 2:**
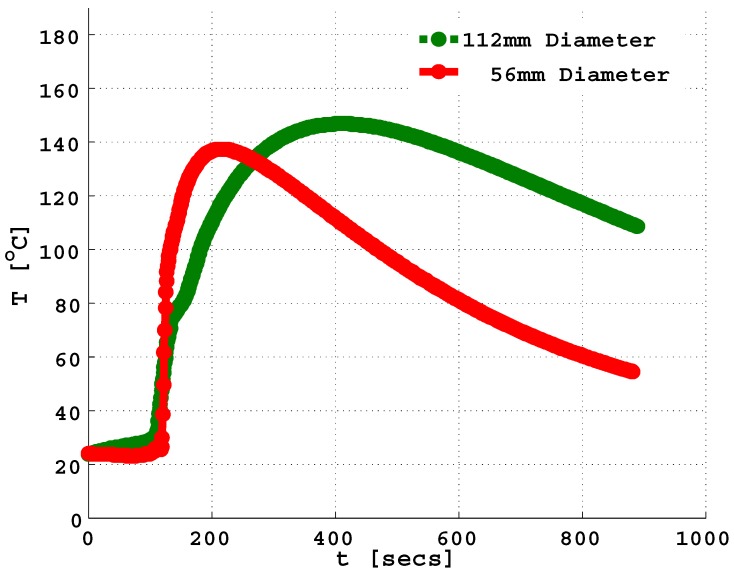
Temperature measurement at a point with equal volumetric reading of 1.25 L from the graduated cylinders. This corresponds to a height of 507.5 mm in the 56 mm diameter tube and 127 mm in the 112 mm diameter tube, respectively.

Results from the free-rise experiments, carried out by our colleagues at the Institute of Lightweight Structures, Chemnitz University of Technology, Germany (Table 1 and Figure 2) reveal a possible influence of geometry constriction on the expanding foams.

The final volume of the expanded PU foam in the 56 mm cylinder was observed to be lower in comparison with that of the 112 mm tube though equal initial masses of 77 and 37 g were injected into each pair of cylinders. This unexpected behavior suggests an interplay between the heat transfer in the reacting system and the proximity of the external environment to the core of the expanding PU foam. In the case where the distance from the core to the bounding surface was small, the rapid heat loss in time (Figure 2) enhanced the reduction in the final volume of the expanded foams.

### 3.2. μCT Scan Analysis

**Figure 3:**
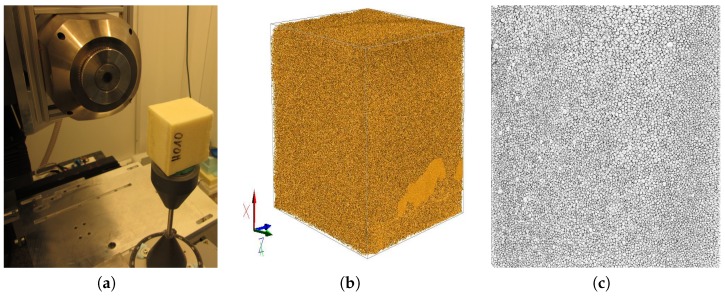
(**a**) Setup for the μCT scan data capture. (**b**) Reconstructed image of the μCT scan raw data using GeoDict. (**c**) Transversal binarised image of CT scan. Slice taken from middle point along the *z*-axis.

To investigate the distribution of solid foam fraction in the matrix, which conversely depicts the void fraction, μCT scan images of a section of the foam with a 50 × 50 × 40 mm dimension from the rectangular mold (Figure 3a) were studied. The corresponding scanned images were reconstructed and analyzed with GeoDict digital material laboratory software [24]. The reconstructed image (Figure 3b) was divided into 15 bins of equal sizes in each direction and the average solid fraction of the foam in each bin was obtained.

**Figure 4:**
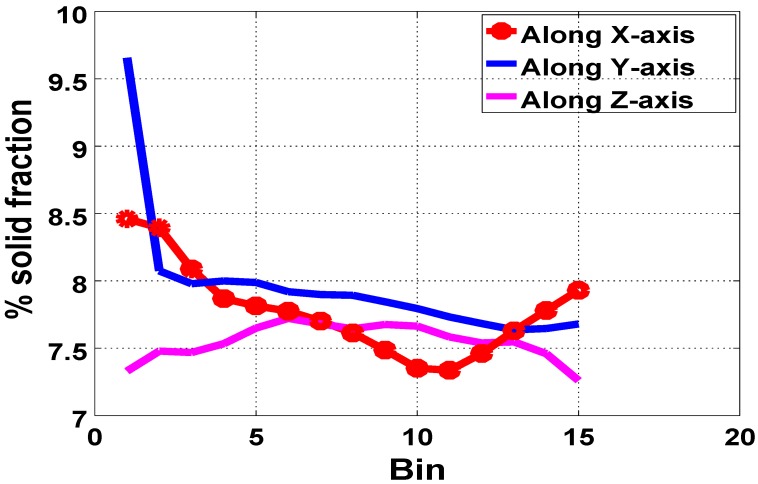
Percentage average volume fraction in each bin along each axis of the reconstructed μCT scan image.

In Figure 4, about 1.2% variation in the solid foam fraction was observed along the *x*-axis, and about 1.8% along the *y*-axis. The *x* and *y*-axis are taken to be perpendicular to the entrant (*y*-*z* plane) and the base (*x*-*z* plane), respectively. However, the distribution of the solid fraction remains almost constant, with a variation of about 0.3%, in the *z*-direction perpendicular to (*x*-*y* plane) from the side wall. We noted that the high solid fractions at the base (Bin 1) along the *y*-axis resulted from the fact that this region got filled first during the injection molding, with some of the material remaining there at lower temperatures throughout the expansion period, thereby resulting in a more dense region in the domain.

Since the proportion of the void in a given material conversely relates to the solid fractions, the distribution of void fractions in the foam matrix, which reflects pore- or bubble-size distribution, is also deductible from Figure 4. More voids were observed toward the core when compared to regions near and at the walls in the expanded foam. Hence, relatively bigger bubbles concentrated at the core when compared to the region near the walls. We therefore surmised that the higher temperature at the core significantly contributes to the expansion rate of the nucleated bubbles in this region. This spatiotemporal variations in temperature and material viscosity induce a nonuniform expansion in the foam, resulting in the variation of bubble size and their distribution across the mold.

In the next section, we numerically explore the reported inhomogeneity observed in the expanded PU foam from experiments, and attempt to address the fundamental questions motivating this study:

- Why does equal mass of PU foam mixture injected into tubes of different dimensions in the injection molding experiment (without overflow) result in expanded foams with different volumes?
- How can this observed nonuniformity be accounted for when modelling and simulating PU foam-expansion processes in other geometries, different from the cylinders?

### 3.3. Numerical Results and Discussion

All numerical implementations in this section were carried out with our inhouse FOAM solver based on the finite-volume method, and resident in the Complex Rheology Simulation (CoRheoS) platform developed at the Fraunhofer ITWM. Unless otherwise stated, all relevant input material values for our simulations were adopted from Reference [19].

#### 3.3.1. Estimating α and Adiabatic Source Term $S_{p}^{ad}$

**Figure 5:**
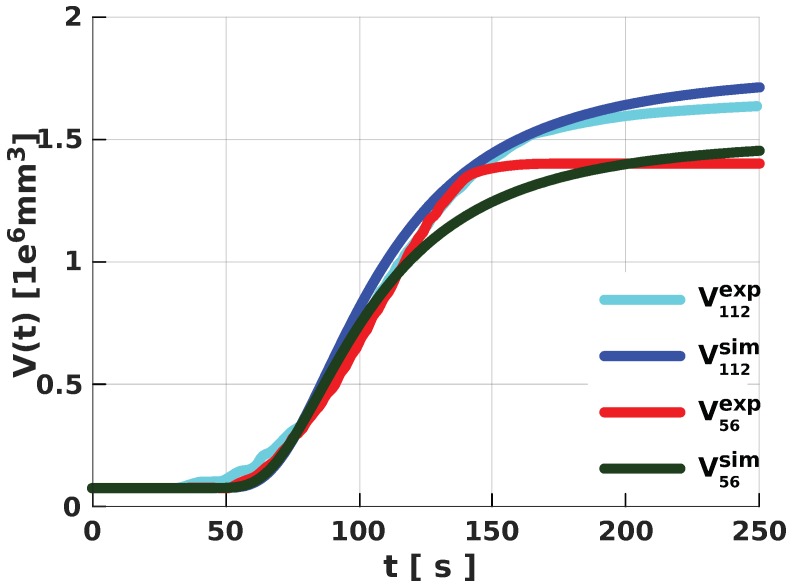
Fit functions for the volume data from the polyurethane (PU) foam-expansion experiments in the 112 mm with fit parameters values $A_{112}=1.7041$, ${\bar{\varepsilon }}_{112}=$ 3,249,603, ${\bar{\pi }}_{112}=3.293393$ and $\gamma_{112}=0.076$, and the 56 mm tubes with parameter values $A_{56}=1.4288$, ${\bar{\varepsilon }}_{56}=$ 3,049,603, ${\bar{\pi }}_{56}=3.3$ and $\gamma_{56}=0.076$.

**Figure 6:**
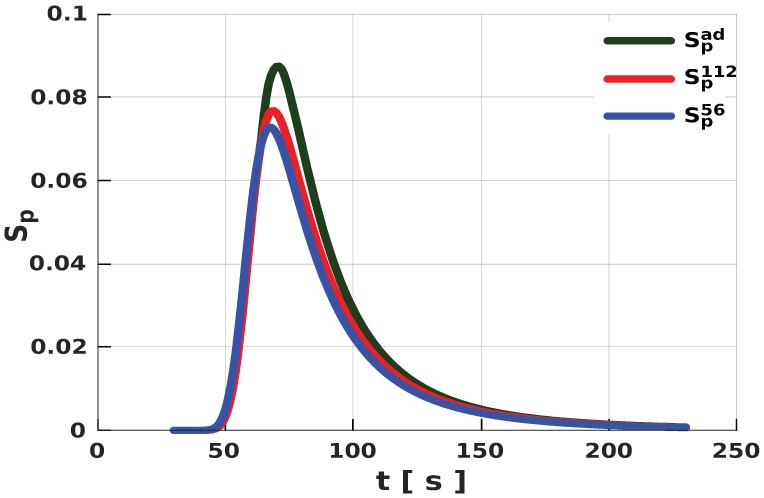
Graphical representation of the expansion source term for each experiment and the estimated $S_{p}^{ad}$ for the adiabatic expansion.

With the volume data from the free-rise expansion experiments in both cylinders, we obtained appropriate parameters for the fit functions (see Equation (4)). Here, fit volumes $V_{112}$ and $V_{56}$ describe the volume of the expanding PU foam in the cylinders with 112 and 56 mm radius, respectively (Figure 5). Following the methodology described in Reference [19], we computed the corresponding $S_{p}^{112}$ for volume $V_{112}$ and $S_{p}^{56}$ for $V_{56}$. Due to the unavailability of the relevant adiabatic measurements for the expanding PU foam, we estimated the value for α and numerically obtained the adiabatic source term $S_{p}^{ad}$ required for our simulations (Figure 6). We conjectured that any given PU foam of the same mixture under adiabatic conditions, irrespective of geometry, would expand with the same volume in time. So, from Equation (12) in the form

(14) $$S_{p}^{b}=\frac{S_{p}^{ad}}{1+\alpha \left(\exp\left(\frac{E_{\mu }}{R}\left(\frac{1}{T^{b}}-\frac{1}{T^{ad}}\right)\right)-1\right)},\;\;\;\;b=\left(112,56\right).$$

and rearranging the resulting expressions, we obtain

(15) $$\alpha =\frac{\hat{S_{p}}-1}{\hat{S_{p}}+\exp\left(\frac{E_{\mu }}{R}\left(\frac{1}{T^{56}}-\frac{1}{T^{ad}}\right)\right)-\hat{S_{p}}\exp\left(\frac{E_{\mu }}{R}\left(\frac{1}{T^{112}}-\frac{1}{T^{ad}}\right)\right)-1},$$

where $\hat{S_{p}}=\frac{S_{p}^{112}}{S_{p}^{56}}$.

Using the simulated average temperatures <T> in each tube over the duration of the free-rise experiment, we obtained α from Equation (15), which varies in time, and computed ${\hat{S}}_{p}^{ad}$ as the average between the $S_{p}^{ad}$ calculated from $S_{p}^{112}$, and that calculated from $S_{p}^{56}$ using the same α, so that

(16) $$S_{p}^{ad}=\langle S_{p}^{112}\Gamma_{\mu }^{112},S_{p}^{56}\Gamma_{\mu }^{56}\rangle ,$$

serve as input to our simulation. With one set of values for the duo, we achieved very good correlation between the volumes from our simulations and those obtained in the free-rise experiments for all considered geometries. However, we noted that approximating $S_{p}^{ad}$ became unnecessary if at least one set of the experimental data was obtained from an adiabatic setup. In such a case, we would only need to obtain the appropriate value for α.

#### 3.3.2. Influence of Thermal Conditions on the Foam-Expansion Process

**Figure 7:**
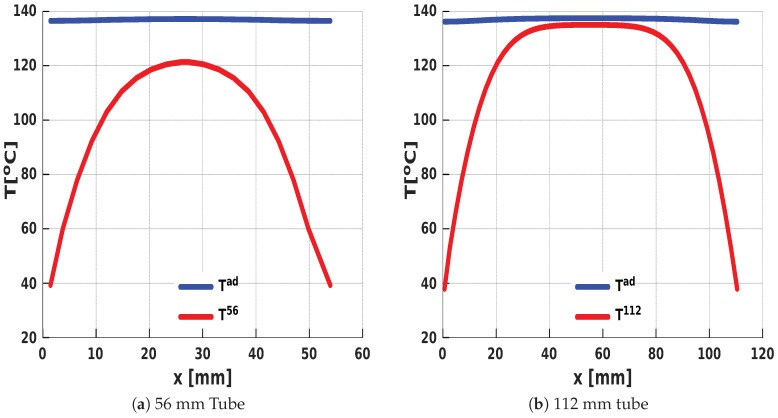
Adiabatic and nonadiabatic temperature profiles along an axis across the tubes (with maximum temperatures in the core) at a fixed time during the expansion process (**a**) in the 56 mm tube and (**b**) in the 112 mm tube.

The significant difference between the adiabatic and nonadiabatic temperatures in the 56 mm tube (Figure 7a) is attributed to the propinquity of the external environment to the core of the expanding foam.

This proximity results in rapid heat loss through the walls of the cylinder due to diffusion. In the 112 mm tube, on the other hand, temperatures at the core were close to adiabatic conditions up to a thermal diffusive layer (near the walls), where heat loss gradually becomes significant (Figure 7b).

**Figure 8:**
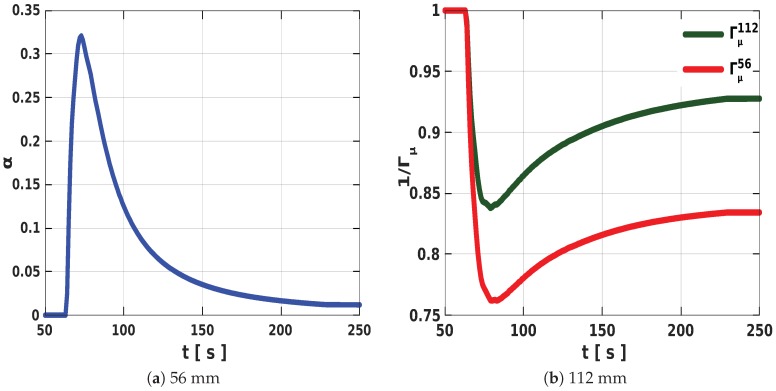
Plots showing the (**a**) variation of the computed α in time and (**b**) the associated damping factor $1/\Gamma_{\mu }$ in each tube as a consequence of thermal disparieties in the cylinders.

The time variation of α in Figure 8a induces a local damping effect (Figure 8b), which is a consequence of the spatial difference in temperatures. Regions with temperature values close to the adiabatic temperature experience minimal or no damping. In addition, depending on the closeness of the bounding surface to the core and the local temperatures in the expanding mixture, the damping effect increases with an increase in α (see Figure 8a,b). Therefore, at higher α values, the mixture in the tube with the 56 mm diameter becomes less expansive when compared with the 112 mm tube, thereby resulting in a significant decrease in the final volume of the expanded foam in the smaller tube.

To further consolidate our proposed mathematical framework for expansion source term $S_{p}$ in Equation (12), we compared the data for the final volumes of the expanded foams from the free-rise experiments with results from our simulations (Table 2). In addition, we simulated a supplementary experimental setup for a free-rise foam-expansion experiment conducted with 77 g of the same PU foam material in a cylindrical tube with 84 mm diameter using the same α and $S_{p}^{ad}$ obtained from our previous calculations (Equations (15) and (16)). The results showed very good correlation with data from experiments (see Table 2).

#### 3.3.3. Spatial Inhomogeneity in the Expanded PU Foams: Simulation Results

**Figure 9:**
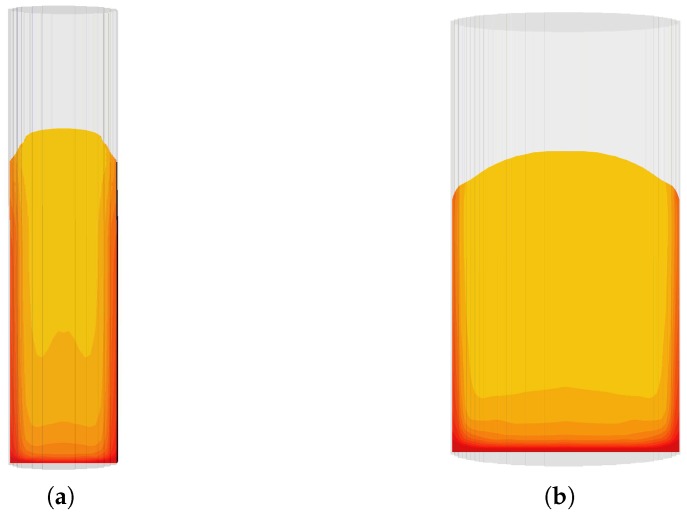
Spatial variation of solid foam fraction in the expanded foam (**a**) in the 56 mm diameter tube and (**b**) in the 112 mm diameter cylinder. The deep red region indicates higher solid fractions.

In our previous study [19], the expanding foam was assumed to undergo nonlocal expansion, which resulted in spatially homogeneous foam fraction within the expanded foam. However, accounting for spatial inhomogeneity in the expansion source term ($S_{p}$) (i.e., α>0), the local variations which arises as a consequence of the spatial changes in temperature and the mixture viscosity influences the distribution of foam fraction in the PU foam system, Figure 9a,b.

This lower temperature at the wall causes an increase in local viscosity. Therefore, the interplay between temperature, viscosity, and local expansion results in restricted growth of the foaming mixture near the walls. This further leads to the formation of densely packed foam fraction around those regions. However, since thermal conditions are higher in the core, the material in this region freely expands.

This corresponds to the lighter colored regions in Figure 9, where foam fractions are lower compared to the deep-red parts with higher solid fractions.

**Figure 10:**
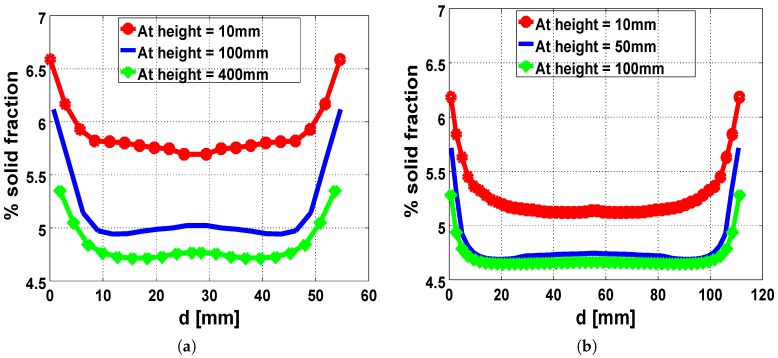
Simulation results showing the symmetric distribution of the solid foam fraction across the cylinder at different heights (**a**) in the 56 mm tube and (**b**) in the 112 mm tube.

The symmetry in flow direction during free-rise expansion in the tubes induces a symmetric distribution of the foam fractions across the tube (Figure 10a,b). More so, the solid fraction of the foamed material was observed to decrease with height around the bounding walls and in the core.

**Figure 11:**
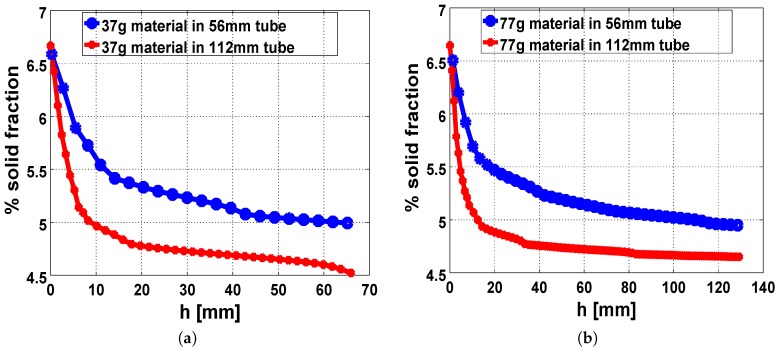
Disparity in foam-fraction distribution from the center of the base to a point in the core for both cylinders, with injected material mass of (**a**) 37 g and (**b**) 77 g.

The observed disparity in the fractions of the expanded foam (Figure 11a,b) directly follows from the temperature conditions in the corresponding setup. Provided the expanding system is non-adiabatic the thickness of the heat diffusion layer near the walls, attributed to the proximity of the core of the expanding foam to the external environment, results in significant increase in the local viscosities around those regions. This diffusion effect is more prominent in the 56 mm cylinder when compared to the 112 mm cylinder. Consequently, the foaming material in the smaller tube experiences more growth restriction around the walls in both cases of the injected masses (37 gm and 77 gm). Therefore, the higher values of the solid foam fractions in the constricted geometry result in denser foam with a reduction in the final volumes of the expanded foams, as seen in the 56 mm cylinder.

**Figure 12:**
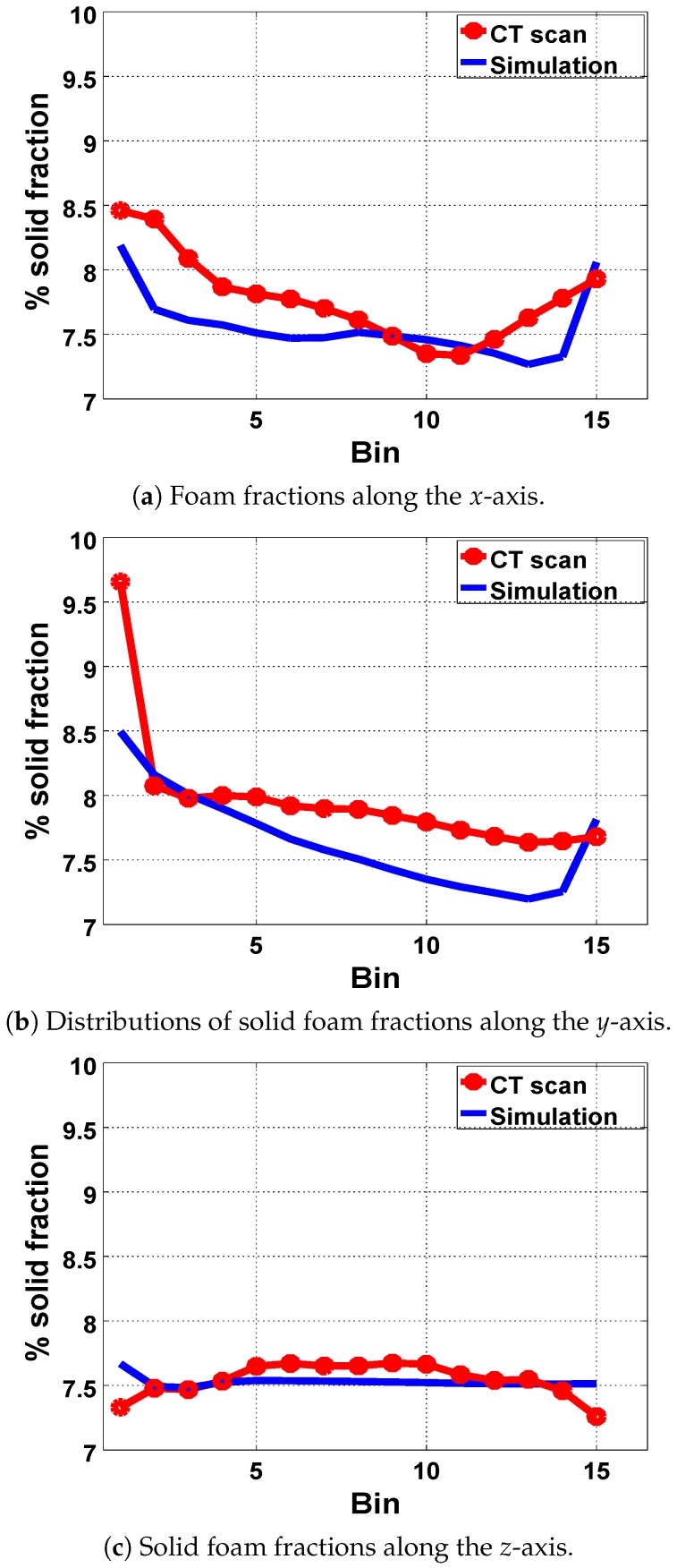
Comparison of the percentage volume fraction from the experiment and those from our simulations along each axis.

Furthermore, we assessed our modelling technique and confirmed its efficacy by validating our results with those from the μCT scan analysis of the expanded foam in the rectangular geometry. Using the same values for α and $S_{p}^{ad}$ obtained from the tube simulations, we simulated the injection molding process of PU foam in a rectangular mold as described in Section 2. We particularly investigated the distribution of the solid fraction and compared our simulation results with the data from μCT scan analysis (Figure 12a–c). In Figure 12a along the *x*-axis, we observed a similar variation (in foam-fraction distribution) of about 1.2% in both cases and, along the *y*-axis, a variation of about 1.3%–1.8% was observed (Figure 12b). A variation of about 0.2%–0.3% was also observed along the *z*-axis, as shown in Figure 12c. The asymmetry observed in the distribution of the solid fractions in Figure 12 is due to the asymmetry in the flow of the reacting mixture along the flow direction during the foam-expansion process. This observation qualitatively agrees with those from the experiments.

**Figure 13:**
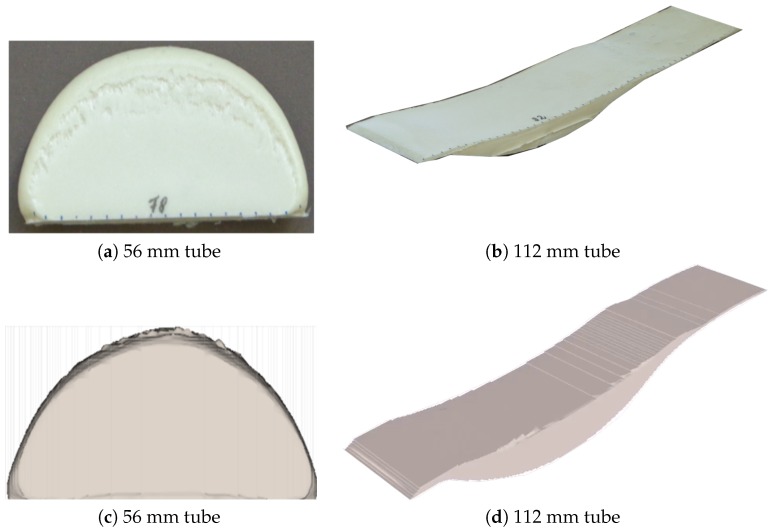
Comparison between simulation and experiment for reaction injection molding in a wavy mold.

Finally, we adopted the obtained models and simulated the reaction injection molding of the PU foams in a wavelike geometry. The comparison between the final results from the simulations and those obtained from the experiment showed very good agreement (Figure 13).

## 4. Conclusions

Inspired by the cell model for a unit bubble expanding in a viscous liquid, we proposed a mathematical framework that accounts for spatial inhomogeneity in a thermally uncontrolled (nonadiabatic) system of expanding polyurethane foam. We surmised that, under adiabatic conditions, a given foam material would expand in the same manner, irrespective of mold geometry. Hence, with the understanding that temperature distribution in a nonadiabatic system plays significant role in the local expansion of the foaming material, the source term driving foam expansion is structured to depend on the variation between adiabatic and nonadiabatic temperatures. With one setup for the expansion source term, calibrated for adiabatic expansions, we studied all flow conditions considered in this work. To understand the degree of nonuniformity in the expanding foam, we modelled and simulated free-rise PU foam-expansion experiments in cylindrical tubes of different diameters, and the injection molding of the same foam in a rectangular mold. A fundamental issue in this study was to investigate the observed difference in the volumes of the expanded PU foam when equal amounts of mass were injected in different geometries.

The interplay between temperature, material viscosity, and the gap between the core and the external environment were observed to affect the distribution of solid foam fractions in the expanded PU foam. In a nonadiabatic system, the thickness of the thermal diffusion layer near the walls resulted in the appearance of a viscous layer that restricts the expansion process around such locations. Then, a densely packed foam fraction formed in the regions near the wall. This phenomenon was more pronounced in constricted geometries, where the core of the expanding foam was relatively closer to the external environment. Therefore, the observed disparity in the volumes of the expanded foam in the free-rise experiments in the cylinders resulted from the combined effects of temperature, viscosity, and the proximity of the foam core to the bounding surface. To validate our models, we compared the results from our simulations with the volume data of the expanded foams, obtained from the free-rise experiments in the cylinders. Our results showed good correlation with those from the experiments.

Images from μCT scans of the expanded foam from the rectangular mold were reconstructed and studied using GeoDict digital material laboratory software. The reconstructed images were analyzed for the distribution of foam fractions in the mold. Analysis showed an average spatial variation of about 1.1% in the foam fraction from the walls to the core of the foam matrix. This observation favorably compared with the results from our injection molding simulation.

This study serves as a platform for our ongoing studies on foam-expansion processes in porous media, with applications in reinforced structures with complex geometry.

## Figures and Tables

**Table 1:** Experiment observations showing the final average volume estimated from several experiments.

Tube Diameter	Injected Mass	Final Volume
(mm)	(g)	1e${}^{6}$ (mm${}^{3}$)
112	77	1.622
56	77	1.401
112	37	0.74547
56	37	0.6452

**Table 2:** Comparison for the final foam volumes from the experiment and our simulations for uniform and nonuniform expansion models using the computed $S_{p}^{ad}$.

		Simulation				
		α=0		α>0		Experiment
d	Mass	Vol Function	Vol	$S_{p}$	Vol	Vol
(mm)	(g)	V(t)	1e${}^{6}$ (mm${}^{3}$)	$S_{p}^{ad}$	1e${}^{6}$ (mm${}^{3}$)	1e${}^{6}$ (mm${}^{3}$)
112	77	$V_{112}$	1.63667	$S_{p}^{ad}$	1.61	1.622
112	37	$V_{112}$	0.813583	$S_{p}^{ad}$	0.7250	0.74547
56	77	$V_{56}$	1.41645	$S_{p}^{ad}$	1.44822	1.401
56	37	$V_{56}$	0.696319	$S_{p}^{ad}$	0.664682	0.6452
		Result from simulation of supplementary experiment				Supplementary Experiment
84	77			$S_{p}^{ad}$	1.55188	1.5657

## Appendix A. Governing Equations

Conservation of Mass

(A1) $$\nabla \cdot v=\frac{1}{V(t)}\frac{dV}{t}=S_{p},$$

Conservation of Momentum

(A2) $$\frac{\partial \rho v}{\partial t}+\nabla \cdot \left(\rho \mathrm{vv}\right)=-\nabla P+\nabla \;\cdot \;\left(\eta_{m}D\right)+\rho g\;,$$

Conservation of Energy

(A3) $$\rho C_{p}\left(\frac{\partial T}{\partial t}+v.\nabla T\right)=\nabla \cdot \left(k\nabla T\right)+\frac{1}{2}\left(\eta_{m}D:D\right)+\rho H_{R}\frac{d\zeta }{dt}.$$

Degree of Polymerization

(A4) $$\frac{d\zeta }{dt}=\left(k_{1}+k_{2}\zeta^{m}\right){\left(1-\zeta \right)}^{n},$$

$k_{1}$, $k_{2}$*m*, and *n* are constants. ζ is the degree of cure/polymerization. ρ is density, *P* is pressure, **D** is deformation tensor, **v** and **g** are, respectively, flow velocity and the gravitational force. $C_{p}$, $H_{R}$, and *k* are the specific heat capacity, heat of reaction, and thermal conductivity, respectively.

## Appendix B. Relationship between Radius *R* and Expansion Source Term *S_p_*

Suppose there are *n* bubbles in the expanding PU foam system, and each bubble of radius R(t) expands uniformly in time with volume V(t). We can write the total volume of the expanding bubbles as

(A5) $$\sum_{i=1}^{n}\frac{1}{V_{i}}\frac{dV_{i}}{dt}=\sum_{i=1}^{n}\frac{3}{4\pi R_{i}^{3}}\frac{d}{dt}\left(\frac{4}{3}\pi R_{i}^{3}\right),$$

since the expansion is uniform, so that $R_{i}^{\prime }s$ are equal in time; then, with some algebraic manipulations, it suffices to write

(A6) $$\frac{1}{V}\frac{dV}{dt}=\frac{3}{R}\frac{dR}{dt}.$$

Therefore,

(A7) $$\left[\frac{1}{V}\frac{dV}{dt}=S_{p}\right]\equiv \frac{3}{R}\frac{dR}{dt}=S_{p}.$$

So that

(A8) $$\frac{1}{R}\frac{dR}{dt}=\frac{1}{3}S_{p}$$
